# Epidemiology and Management of Proximal Femoral Fractures in Italy between 2001 and 2016 in Older Adults: Analysis of the National Discharge Registry

**DOI:** 10.3390/ijerph192416985

**Published:** 2022-12-17

**Authors:** Umile Giuseppe Longo, Marco Viganò, Laura de Girolamo, Giuseppe Banfi, Giuseppe Salvatore, Vincenzo Denaro

**Affiliations:** 1 Research Unit of Orthopaedic and Trauma Surgery, Fondazione Policlinico Universitario Campus Bio-Medico, Via Alvaro del Portillo, 200, 00128 Roma, Italy; 2 Research Unit of Orthopaedic and Trauma Surgery, Department of Medicine and Surgery, Università Campus Bio-Medico di Roma, Via Alvaro del Portillo, 21, 00128 Rome, Italy; 3 IRCCS Istituto Ortopedico Galeazzi, Via Riccardo Galeazzi 4, 20161 Milano, Italy

**Keywords:** hip fractures, epidemiology, internal fixation, hemiarthroplasty, fracture management

## Abstract

This study aims to determine the annual incidence of proximal femoral fractures in Italy in the period between 2001 and 2016 among older adults, and to describe the trends in the clinical management of these cases. Data were retrieved from the National Hospital Discharge records issued by the Italian Ministry of Health and from the Italian Institute for Statistics. The number of hospitalizations increased between 2001 and 2016, while the age-adjusted yearly incidence decreased from 832.2 per 100,000 individuals to 706.2. The median age was 83 years (IQR 78–88) with a large majority of females (76.6%). The type of fracture varied with age in female subjects, with older women more frequently reporting pertrochanteric fractures. Therapeutic strategies for the different types of fracture depended on patients’ age. During the study years, improvements in fracture classification and management strategies were observed, with a clear decreasing trend for non-operative solutions. In conclusion, the number of proximal femur fractures in older adults is growing, even if at a lower rate compared to population aging. The Italian surgical practice changed during the study period towards the implementation of the most recent guidelines.

## 1. Introduction

Fractures of the proximal femur are common events in older adults that lead to significant morbidity and disability [1]. In the absence of specific contra-indication, rapid surgical treatment is advised, but the type of intervention may significantly vary based on patient’s characteristics, and therefore indications are still disputed [1,2,3]. Recently, total hip arthroplasty (THA) gained popularity, with guidelines suggesting this treatment as the optimal treatment in active patients [4]. Nevertheless, internal fixation (IF) and hemiarthroplasties (HA) are historically the most common surgical solutions, and they still represent the most frequently applied treatments [5]. In specific cases, presenting significant comorbidities, a non-operative management is considered [6]. Given the high incidence of these fractures in subjects older than 65 years, and given the population-aging phenomenon, the management of proximal femoral fractures represents an increasingly important topic for national healthcare systems worldwide, as well as for the orthopedic community. Several reports from different countries highlighted an increase in the total number of proximal femoral fractures [7,8,9,10,11,12,13,14,15], even if the age-adjusted incidence is reported to be stable or decreasing [8,16,17,18,19,20,21,22]. The choice of treatment and other aspects such as the time interval between injury and surgery may significantly influence the clinical outcome [23,24,25], and thus the investigation of these topics would be of paramount importance for the future planning of proximal femoral fractures management.

The present study aims to determine the annual incidence of proximal femoral fractures in the older Italian population between 2001 and 2016, as well as to describe the trends in the clinical management of these patients in order to provide a review of the past activity and hints about possible future scenarios. Data were provided by the Italian Ministry of Health. The dataset reported only index hospitalization for proximal femoral fracture, and thus no additional information about the patients’ status, comorbidities and outcomes were available. In addition, the ICD-9-CM codification (2015 version) was used. Since it does not include a specific classification for displaced/non-displaced fracture and stable/unstable fractures, this information was not included in the analysis.

## 2. Materials and Methods

### 2.1. Data Collection

The manuscript was prepared following STROBE guidelines [26]. Data were retrieved from the National Hospital Discharge records (Scheda di Dimissione Ospedaliera, SDO) issued between 2001 and 2016. These anonymous data were initially collected by the Italian Ministry of Health and were then made available to the authors. The database includes the following information: age, sex, length of the hospitalization, public or private reimbursement, primary and secondary diagnoses, and primary and secondary interventions. A second database was used to retrieve data about the general population at the national level; these data were obtained from the Italian National Institute for Statistics (ISTAT) website [27]. These data report the total number of subjects living in Italy each year, with a breakdown by age and gender. A reliability analysis was performed by the statistician in charge of the analysis in regard to the hospitalization dataset: validity was evaluated by checking the format and range of values for each variable, and no issues were identified. Completeness was checked and all records contained the minimum entries to be considered in the analysis. A duplicate search was performed to test the uniqueness of each record, and no duplicates were found. The dataset provided by ISTAT respected the National Institute Standard of Quality [28].

**Figure 1:**
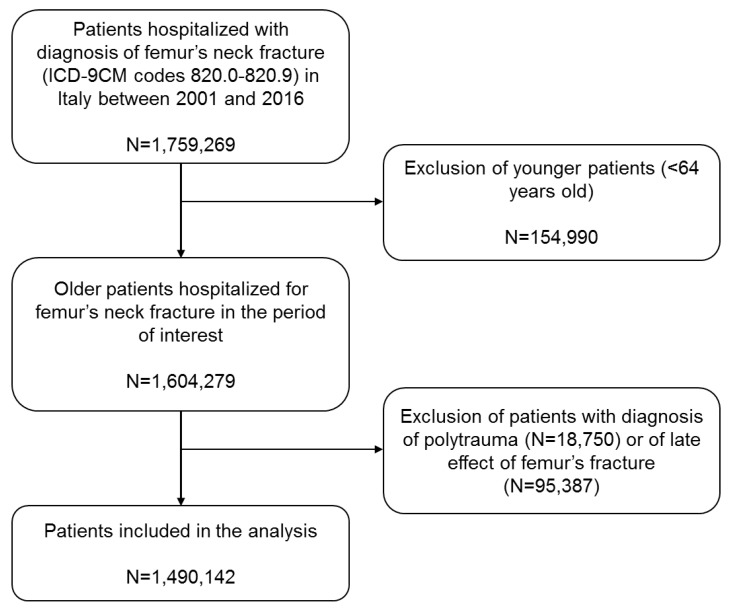
Flow chart of patients’ selection process.

The inclusion criteria was hospitalization in Italy with a diagnosis of fractures of the proximal femur, identified based on code 820.0–820.9 of the International Classification of Diseases, Ninth Revision, Clinical Modification (ICD-9-CM) between 2001 and 2016. The exclusion criteria were the following: patients hospitalized in other countries, age <65 years old, diagnosis of polytrauma and diagnosis of a late effect of fracture of the proximal femur or lower extremity (ICD codes 905.3 and 905.4). A flow chart of the patient selection process is reported in Figure 1.

### 2.2. Statistical Analysis

The analyses were performed using SAS software v9.4 (SAS Institute, Cary, NC, USA). Data concerning categorical variables are reported as counts and percentages, while continuous data are reported as median and interquartile range (IQR), unless otherwise indicated. The raw incidence per year was calculated as the number of events divided by the number of people living in Italy in the year of interest, and it was reported as the relative frequency per 100,000 individuals. Age-adjusted incidence was calculated after normalizing the year-specific population by age category. In particular, the sum of persons at risk per year in each age category was divided by the total number of persons at risk during the study period to obtain the mean weight of each age category in the analyzed population. Then, the yearly age-specific incidence was calculated by dividing the number of events in each age category by the correspondent number of people at risk per year. This ratio was multiplied by the weight of the age category in the overall population, and then the sum of the numbers obtained for each age category was calculated to obtain the year-specific age-adjusted incidence. Supplementary Table S1 reports the numbers and calculations. Differences among proportions were assessed using either Fisher’s exact test or the proportion trend test. Linear regression was used to evaluate the trend in total number of events during the study years.

## 3. Results

### 3.1. Total Number and Incidence of Hospitalizations in the Analyzed Period

**Figure 2:**
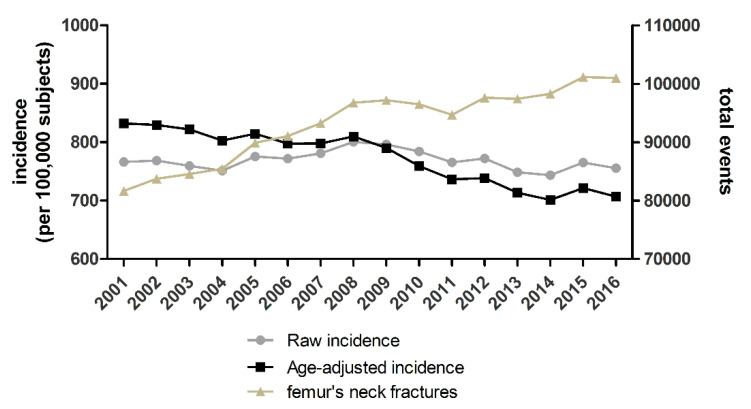
Left y-axis: raw incidence and age-adjusted incidence (events/100,000 adults >65 y/o) of hospitalization for proximal femoral fracture per year. Right y-axis: total number of fractures of the proximal femur per year.

In the analyzed period, 1,490,142 hospitalizations for the diagnosis of proximal femoral fracture were recorded among the +65 y/o Italian population, with an overall incidence of 769.7 events per 100,000 person-years. The total number of hospitalizations showed an increasing trend between 2001 and 2016 from 81,648 to 100,998 (Figure 2). This increase was linear (R^2^ = 0.899), with a mean increase (slope) of 1261 events per year (CI95%: 1019–1503). On the other hand, the raw incidence slightly decreased between 2001 and 2016, from 766 to 755 events per 100,000 person-years, with a peak of 800 in 2008. Interestingly, in the same time-period, the age-adjusted yearly incidence showed a significantly decreasing trend (*p* < 0.001) from 832 hospitalizations per 100,000 individuals in 2001 to 707 in 2016. (Figure 2). The median age of patients hospitalized with a diagnosis of proximal femoral fracture was 83 years (IQR 78–88). Indeed, the incidence of this diagnosis exponentially increased with age, from 145.3 events per 100,000 person-years among patients aged 65–69 years old up to 3563 in subjects aged 95–99 y/o. The vast majority of subjects, 1,141,716 (76.6%), were females (Table 1).

### 3.2. Type of Fracture Differs Based on Gender and Age

Closed pertrochanteric fractures (icd9 code 820.2x) were the most frequently observed, representing 51.1% of the total events, especially a closed fracture of the trochanteric section of the neck of the femur (code 820.20, 34.2% of total events). Closed transcervical fractures were observed in 33.1% of the analyzed hospitalizations, with code 820.01 (closed upper transcervical fracture) the most frequent among the intracapsular fractures (14.3% of total events). Open fractures accounted for only 1.7% of events (0.8% transcervical, 0.9 pertrochanteric). The total number and frequency of the different types of fractures in the dataset are reported in Table 2.

**Figure 3:**
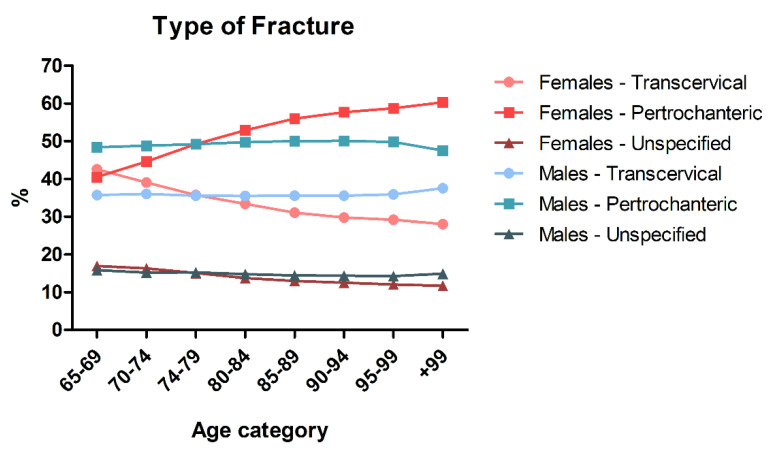
Relative frequency of types of fracture in males and females in different age categories.

The types of fractures are evenly distributed among the different age classes for male patients, while age is significantly associated with the type of fractures in female subjects. In particular, transcervical fractures are more frequent in younger females (41.5% among the 65–69 y/o and 27.6%), while older women experience pertrochanteric fractures at a higher rate (59.3% in the 99+ y/o category vs. 39.7% in 65–69 y/o) (Figure 3). Complete data are reported in Supplementary Table S2.

During the 15 years of interest, a constant decreasing trend for “unspecified” fractures was observed, starting from 25.2% of cases in 2001 to 11.8% in 2016. At the same time, increases in specific diagnoses were observed, in particular for what concern upper transcervical fractures (820.01), from 6.2% to 15.6%. Notably, closed basicervical fractures steadily decreased during the study years (11.3% in 2001, 5.8% in 2016) (Supplementary Table S2).

### 3.3. Fracture Management in Different Patients and Types of Fracture

A total of 20.3 % of hospitalizations for proximal femoral fracture were not associated with surgeries or reductions.

Transcervical fractures (open or closed) were more frequently treated by HA (49.7% of cases) or THA (17.5%) compared to open or closed reduction with internal fixation (14.5%). Specifically, basicervical fractures were treated by HA and THA less frequently compared to upper cervical and midcervical fractures, while reduction with internal fixation or non-surgical management were applied more frequently. HA was performed more frequently in older individuals (>55.0% in subjects >80 years old vs. 20.0% in 65–69 y/o), while the contrary was observed for THA (41.7% in 65–69 y/o, <10.0% above 85 y/o).

**Figure 4:**
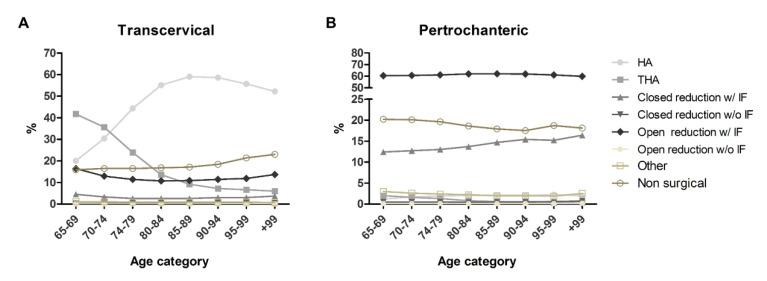
Relative frequency of treatments for transcervical (**A**) and pertrochanteric (**B**) fractures in different age categories. HA, hemiarthroplasty; THA, total hip arthroplasty; w/ IF, with internal fixation; w/o IF, without internal fixation.

Pertrochanteric fractures were frequently treated with an open or closed reduction with internal fixation (75.7% of cases), with HA and THA representing rare treatment choices (2.0% and 0.9%, respectively). Differences were observed in the management of intratrochanteric fractures compared to trochanteric and subtrochanteric fractures, with a higher frequency of closed reduction with internal fixation (22.4% vs. 11.5% and 11.6%, respectively) and a lower frequency of open reduction with internal fixation (55.4% vs. 63.3% and 67.4%, respectively). Table 3 reports the absolute and relative frequency of each management strategy for the different types of fractures. Younger patients underwent closed reduction with internal fixation less frequently than older subjects (12.4% in 65–69 y/o, up to 16.4% in +99 y/o). Open reduction with internal fixation showed a similar frequency among different age categories. Figure 4 shows the treatment choices in different age classes and type of fractures.

Gender does not influence the treatment choice, even if a higher percentage of non-surgical treatment was recorded for males compared to females, independently from the type of fracture. Supplementary Table S3 reports the specific frequencies of surgical treatment in different age categories and types of fractures. Reductions without fixation are rare treatment choices, reported with a frequency <1%.

### 3.4. Trends in Treatment Strategies during Study Years

**Figure 5:**
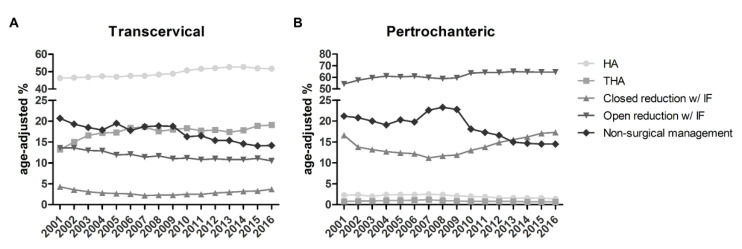
Age-adjusted relative frequency of treatments of transcervical (**A**) and pertrochanteric (**B**) fractures between 2001 and 2016. HA, hemiarthroplasty; THA, total hip arthroplasty; w/ IF, with internal fixation; w/o IF, without internal fixation.

During the 15 study years, changes in the management choices were observed for both pertrochanteric and transcervical fractures. Age-adjusted estimations were obtained in order to account for the variability in treatment choice among patients of different age, and are expressed as treatments per 100 events. While HA was the main choice for the treatment of transcervical fractures in the whole period, its application slightly increased from 46.3 to 51.7 between 2001 and 2016. The same was observed for THA, increasing from 13.2 to 19.1. Conversely, other solutions, including closed and open reduction with internal fixation or non-surgical management, decreased during the study years (Figure 5A).

Considering pertrochanteric fractures, an increasing trend was observed in the incidence of open reduction with internal fixation, starting from 54.3 surgeries in 2001 to 64.6 surgeries per 100 events in 2016. Again non-surgical solutions dropped from 21.2 in 2001 to 14.5 choices per 100 events in 2016, while closed reduction with internal fixation remained similar between the first and last study years (2001:16.6; 2016:17.3) with a reduction observed between 2002 and 2009 with an incidence of less than 12.0 surgeries per 100 pertrochanteric fractures. HA and THA were already rare in 2001 and their incidence in this type of fracture further reduced during the study years (Figure 5B). Supplementary Table S4 reports the incidence of each treatment over the study period.

### 3.5. Length of Hospitalization

The length of hospitalization showed a median of 12 days, with an interquartile range of 9–18 days. A progressive reduction in this parameter was observed in the median length of hospitalization from 14 (IQR: 9–20) days in 2001 to 11 (8–16) days in 2016. Similar hospitalization lengths were observed in males (12, IQR 8–19) and females (12, IQR 9–19) and in different age classes, with a minimum median of 11 (IQR 7–16) in patients older than 99 y/o and a maximum of 13 (IQR 9–18) in patients 74–84 y/o. Again, no differences were observed between pertrochanteric and transcervical fractures, with medians equal to 12 days in both cases and slightly different IQR (8–18 and 9–17, respectively).

### 3.6. Associated Diagnosis

The most frequent associated diagnoses reported for these hospitalization were acute posthemorrhagic anemia (19.7%), hypertension (17.5%), heart disease (8.9%) and diabetes (8.3%). Table 4 reports the demographic data in detail. In general, acute posthemorragic anemia, hypertension and osteoporosis were more frequent among female subjects, while heart, respiratory and Parkinson’s diseases were more frequent among males.

## 4. Discussion

The present study shows that proximal femoral fractures in Italy are increasing in number and that the choice for operative solutions became more frequent over non-surgical management between 2001 and 2016.

The increase in total numbers of proximal femoral fractures appears strictly related to the population ageing, since the age-adjusted incidence has decreased in the same period. This appears to be a common phenomenon worldwide [8,16], possibly due to the reduction in post-fracture mortality rate, as well as the implementation of policies aimed at preventing osteoporosis [22,29,30,31,32]. Nevertheless, the increase in the overall number of fractures suggests that the magnitude of this decrease is insufficient to compensate for the effect of population ageing [11,33]. Indeed, the increase in the absolute numbers of proximal femoral fractures, as well as the higher incidence of these events in females and older subjects, are consistent with the evidence provided by several authors from different countries [7,9,10,12,13,15,34]. The present study confirms these findings in a larger cohort (entire Italian population) and considers a longer time-period (16 years) compared to most of the previous studies.

During the study period, an increasing trend towards the use of surgical solutions has been observed in both pertrochanteric and transcervical fractures. In addition, the incidence of specific surgeries (hemi- and total arthroplasties for transcervical fractures and closed/open reduction with internal fixation for pertrochanteric fractures) grew over time, suggesting an optimization of management strategies towards the adherence to the most recent guidelines [35]. Indeed, the growing incidence of THA observed in Italy for the treatment of transcervical fractures is consistent with reports from Canada, Australia, South Korea, Finland and the United States [36,37,38,39,40,41]. The use of THA is recommended by the AAOS practical guidelines [35], even if it does not provide advantages compared to HA in older patients [42]. Nevertheless, this mainly applies to relatively younger and healthy patients thus limiting its use [43]. In the Italian cohort, THA was the main treatment choice in subjects younger than 74 years old for transcervical fractures. Aside from the type of fracture and the age of the patient, it should be considered that the management of proximal femoral fractures may depend on external factors, such as the surgeon’s specific expertise, the volumes of specific procedures usually performed at the hospital and insurance, with patients with private insurance undergoing THA more frequently than those without [44,45].

The length of hospitalization progressively decreased during the study period. This could be due to the effects of two distinct policies, one focused on cost reduction and shortening post-operative hospitalization [46], and the other aimed at reducing the time between hospitalization and surgery. A reduced time to surgery provides better results in terms of survival and clinical outcomes [47,48], while the reduced hospitalization time after surgery did not produce negative effects on post-operative mortality rate [49]. Unfortunately, no data were available regarding the time interval between hospitalization and surgery in the analyzed cohort, and thus we cannot confirm the effect of this parameter on total hospitalization length in the Italian cohort. The same decreasing trend in the length of hospitalization has been reported by other authors, suggesting the existence of a global trend [49,50].

Given the high median age of patients, severe co-morbidities are frequent in patients suffering from proximal femoral fractures. In our cohort, hypertension, heart disease and diabetes were frequent associate diagnoses, while the high incidence of post-hemorrhagic anemia may represent a direct consequence of the injury and/or the treatment. The frequency of these pathologies is comparable to that observed in similar patients from Denmark, where these associated diagnoses were also identified as risk factors for 1-year mortality after hospitalization for proximal femoral fracture [49]. A relevant percentage of patients (5.8%) was affected by dementia or Alzheimer disease, conditions that are known to require special attention [51]. Osteoporosis appears to be under-diagnosed in this cohort, possibly due to a lack of interest in reporting this associated diagnosis from the perspective of the healthcare providers.

Interestingly, the “unspecified” type of fracture decreased significantly during the time period, and this is suggestive of improvements in diagnostic techniques over time [52,53]. In addition, the rate of basicervical fractures, whose diagnosis is often difficult [54], dropped from 11.3% in 2001 to 5.8% in 2016, and this is possibly an indicator of a decreased rate of the misdiagnosis of upper perthrocanteric fractures as basicervical fractures. This possible bias should be considered when comparing a series of cases between different decades.

The present study has limitations. The main limitation is the reliance on administrative data, not allowing for the evaluation of outcomes such as mortality and relapses, and accounting for a certain amount of missing information, as confirmed by the large percentage of the unspecified type of fracture observed in the sample, especially in the early 2000s. Unfortunately, the ICD-9 classification used in the dataset (2015 version) does not distinguish between displaced and non-displaced fractures, thus limiting the description of these fracture types’ incidence and management. In addition, the dataset contains only index hospitalizations and, thus, the associated diagnosis is not representative of comorbidities, since their inclusion in the records is at the discretion of the operator.

## 5. Conclusions

In conclusion, the number of fractures of the proximal femur in older adults grew in the analyzed period, even if at a lower rate compared to what would be expected based on the increase in population age. The surgical approach changed during the study period following the implementation of up-to-date guidelines.

## Figures and Tables

**Table 1:** Patients’ demographics.

	Males	Females	Total
Events	348,426 (23.4%)	1,141,716 (76.6%)	1,490,142
Age, years (Median, IQR)	82 (77–87)	83 (78–88)	83 (78–88)

**Table 2:** Specific diagnosis per year.

	820.01	820.02	820.03	820.11	820.12	820.13	820.20	820.21	820.22	820.30	820.31	820.32	Unspecified
2001	5091 (6.2%)	6583 (8.1%)	9206 (11.3%)	78 (0.1%)	324 (0.4%)	348 (0.4%)	25,643 (31.4%)	10,149 (12.4%)	2909 (3.6%)	345 (0.4%)	219 (0.3%)	211 (0.3%)	20,542 (25.2%)
2002	7855 (9.4%)	8186 (9.8%)	9551 (11.4%)	69 (0.1%)	296 (0.4%)	298 (0.4%)	26,478 (31.6%)	10,512 (12.5%)	3473 (4.1%)	257 (0.3%)	138 (0.2%)	202 (0.2%)	16,448 (19.6%)
2003	10,345 (12.2%)	8559 (10.1%)	8643 (10.2%)	35 (0%)	269 (0.3%)	267 (0.3%)	28,361 (33.5%)	9557 (11.3%)	3774 (4.5%)	240 (0.3%)	110 (0.1%)	129 (0.2%)	14,250 (16.9%)
2004	11,445 (13.4%)	8868 (10.4%)	8303 (9.7%)	42 (0%)	248 (0.3%)	228 (0.3%)	29,277 (34.3%)	9016 (10.5%)	4019 (4.7%)	229 (0.3%)	120 (0.1%)	154 (0.2%)	13,512 (15.8%)
2005	12,965 (14.4%)	9717 (10.8%)	8345 (9.3%)	52 (0.1%)	249 (0.3%)	206 (0.2%)	30,535 (34%)	9539 (10.6%)	4275 (4.8%)	314 (0.3%)	145 (0.2%)	147 (0.2%)	13,406 (14.9%)
2006	13,490 (14.8%)	9697 (10.7%)	8103 (8.9%)	35 (0%)	198 (0.2%)	209 (0.2%)	31,438 (34.5%)	9667 (10.6%)	4569 (5%)	271 (0.3%)	211 (0.2%)	158 (0.2%)	12,991 (14.3%)
2007	13,855 (14.9%)	10,107 (10.8%)	7724 (8.3%)	51 (0.1%)	253 (0.3%)	254 (0.3%)	32,344 (34.7%)	10,161 (10.9%)	5101 (5.5%)	305 (0.3%)	231 (0.2%)	171 (0.2%)	12,703 (13.6%)
2008	13,978 (14.4%)	10,269 (10.6%)	7961 (8.2%)	49 (0.1%)	302 (0.3%)	321 (0.3%)	33,799 (34.9%)	10,741 (11.1%)	5173 (5.3%)	369 (0.4%)	266 (0.3%)	188 (0.2%)	13,323 (13.8%)
2009	14,038 (14.4%)	10,518 (10,8%)	7432 (7.6%)	45 (0%)	360 (0.4%)	251 (0.3%)	34,103 (35.1%)	11,352 (11.7%)	5369 (5.5%)	433 (0.4%)	309 (0.3%)	185 (0.2%)	12,786 (13.2%)
2010	14,771 (15.3%)	11,005 (11.4%)	6871 (7.1%)	76 (0.1%)	413 (0.4%)	186 (0.2%)	34,333 (35.6%)	10,657 (11%)	5596 (5.8%)	396 (0.4%)	312 (0.3%)	153 (0.2%)	11,704 (12.1%)
2011	15,075 (15.9%)	10,964 (11.6%)	6685 (7.1%)	117 (0.1%)	435 (0.5%)	165 (0.2%)	33,158 (35%)	10,583 (11.2%)	5457 (5.8%)	312 (0.3%)	357 (0.4%)	165 (0.2%)	11,225 (11.9%)
2012	15,512 (15.9%)	10,739 (11%)	6671 (6.8%)	144 (0.1%)	534 (0.5%)	198 (0.2%)	34,192 (35%)	11,737 (12%)	5400 (5.5%)	306 (0.3%)	393 (0.4%)	185 (0.2%)	11,593 (11.9%)
2013	15,733 (16.2%)	11,360 (11.7%)	6297 (6.5%)	170 (0.2%)	556 (0.6%)	160 (0.2%)	33,698 (34.6%)	11,988 (12.3%)	5386 (5.5%)	332 (0.3%)	361 (0.4%)	146 (0.1%)	11,214 (11.5%)
2014	16,177 (16.5%)	11,238 (11.4%)	6155 (6.3%)	109 (0.1%)	717 (0.7%)	176 (0.2%)	33,930 (34.5%)	11,966 (12.2%)	5511 (5.6%)	301 (0.3%)	523 (0.5%)	172 (0.2%)	11,322 (11.5%)
2015	16,430 (16.2%)	11,677 (11.5%)	6051 (6%)	144 (0.1%)	808 (0.8%)	234 (0.2%)	34,033 (33.6%)	13,152 (13%)	5939 (5.9%)	370 (0.4%)	564 (0.6%)	208 (0.2%)	11,538 (11.4%)
2016	15,748 (15.6%)	11,760 (11.6%)	5867 (5.8%)	242 (0.2%)	809 (0.8%)	283 (0.3%)	34,177 (33.8%)	13,338 (13.2%)	5620 (5.6%)	419 (0.4%)	594 (0.6%)	239 (0.2%)	11,902 (11.8%)

Data are reported as absolute frequency and row percentage for each year: 820.01, closed upper transcervical fracture; 820.02, closed midcervical fracture; 820.03, closed basicervical fracture; 820.11, open upper transcervical fracture; 820.12, open midcervical fracture; 820.13, basicervical fracture; 820.20, closed trochanteric fracture; 820.21, closed intratrochanteric fracture; 820.22, closed subtrochanteric fracture; 820.30, open trochanteric fracture; 820.31, opem intratrochanteric fracture; 820.32, open subtrochanteric fracture.

**Table 3:** Type of fracture and therapeutic strategy.

	HA	THA	CRwIF	CRw/oIF	ORwIF	ORw/oIF	Other	Non-Surgical
82,001	117,300 (55.2%)	43,928 (20.7%)	2706 (1.3%)	394 (0.2%)	14,297 (6.7%)	18 (0%)	1491 (0.7%)	32,374 (15.2%)
82,002	85,760 (53.2%)	30,232 (18.7%)	4963 (3.1%)	271 (0.2%)	13,817 (8.6%)	28 (0%)	1414 (0.9%)	24,762 (15.4%)
82,003	42,792 (35.7%)	12,651 (10.6%)	6220 (5.2%)	822 (0.7%)	29,366 (24.5%)	61 (0.1%)	1643 (1.4%)	26,310 (21.9%)
82,011	694 (47.6%)	194 (13.3%)	52 (3.6%)	2 (0.1%)	188 (12.9%)	1 (0.1%)	27 (1.9%)	300 (20.6%)
82,012	3727 (55%)	1095 (16.2%)	366 (5.4%)	18 (0.3%)	444 (6.6%)	0 (0%)	60 (0.9%)	1061 (15.7%)
82,013	954 (25.2%)	346 (9.1%)	269 (7.1%)	18 (0.5%)	578 (15.3%)	2 (0.1%)	38 (1%)	1579 (41.7%)
82,020	10,670 (2.1%)	4724 (0.9%)	58,525 (11.5%)	2542 (0.5%)	322,283 (63.3%)	452 (0.1%)	12,134 (2.4%)	98,169 (19.3%)
82,021	2892 (1.7%)	1057 (0.6%)	39,046 (22.4%)	656 (0.4%)	96,433 (55.4%)	154 (0.1%)	3059 (1.8%)	30,818 (17.7%)
82,022	1650 (2.1%)	769 (1%)	8967 (11.6%)	374 (0.5%)	52,284 (67.4%)	67 (0.1%)	1747 (2.3%)	11,713 (15.1%)
82,030	231 (4.4%)	95 (1.8%)	565 (10.9%)	21 (0.4%)	2108 (40.5%)	5 (0.1%)	186 (3.6%)	1988 (38.2%)
82,031	81 (1.7%)	43 (0.9%)	1164 (24%)	99 (2%)	2634 (54.3%)	9 (0.2%)	104 (2.1%)	719 (14.8%)
82,032	56 (2%)	41 (1.5%)	458 (16.3%)	18 (0.6%)	1332 (47.4%)	10 (0.4%)	86 (3.1%)	812 (28.9%)
unspecified	79,621 (37.8%)	26,885 (12.8%)	8113 (3.9%)	683 (0.3%)	20,301 (9.6%)	114 (0.1%)	2654 (1.3%)	72,088 (34.3%)

Data are reported as absolute frequency and row percentage for each type of fracture: 82,001, closed upper transcervical fracture; 82,002, closed midcervical fracture; 82,003, closed basicervical fracture; 82,011, open upper transcervical fracture; 82,012, open midcervical fracture; 82,013, basicervical fracture; 82,020, closed trochanteric fracture; 82,021, closed intratrochanteric fracture; 82,022, closed subtrochanteric fracture; 82,030, open trochanteric fracture; 82,031, open intratrochanteric fracture; 82,032, open subtrochanteric fracture. HA, hemiarthroplasty; THA, total hip arthroplasty; CRwIF, closed reduction with internal fixation; CRw/oIF, closed reduction without internal fixation; ORwIF, open reduction with internal fixation; ORw/oIF, open reduction without internal fixation.

**Table 4:** Diagnosis associated with proximal femoral fractures.

Associated Diagnosis			
Acute Posthemorrhagic Anemia	16.3%	20.7%	19.7%
Hypertension	14.9%	18.2%	17.5%
Heart Disease	11.3%	8.2%	8.9%
Diabetes	8.3%	8.3%	8.3%
Dementia	5.7%	5.8%	5.8%
Respiratory Disease	7.0%	2.8%	3.8%
Osteoporosis	1.1%	2.3%	2.1%
Parkinson Disease	2.7%	1.4%	1.6%

## Data Availability

No new data were created or analyzed in this study. Data sharing is not applicable to this article.

## Supplementary Materials

The following supporting information can be downloaded at: https://www.mdpi.com/article/10.3390/ijerph192416985/s1, Table S1: Calculation of total events, raw incidence and age-adjusted incidence; Table S2: Type of fracture and therapeutic strategy; Table S3: Therapeutic management in different types of fracture and age categories; Table S4: Incidence of different treatments during the study years in pertrochanteric and transcervical fractures.

## References

[1] Florschutz A.V., Langford J.R., Haidukewych G.J., Koval K.J. (2015). Femoral Neck Fractures: Current Management. J. Orthop. Trauma.

[2] Forsh D.A., Ferguson T.A. (2012). Contemporary Management of Femoral Neck Fractures: The Young and the Old. Curr. Rev. Musculoskelet Med..

[3] Guyen O. (2019). Hemiarthroplasty or Total Hip Arthroplasty in Recent Femoral Neck Fractures?. Orthop. Traumatol. Surg. Res..

[4] Roberts K.C., Brox W.T., Jevsevar D.S., Sevarino K. (2015). Management of Hip Fractures in the Elderly. JAAOS-J. Am. Acad. Orthop. Surg..

[5] Lutnick E., Kang J., Freccero D.M. (2020). Surgical Treatment of Femoral Neck Fractures: A Brief Review. Geriatrics.

[6] Ko F.C., Morrison R.S. (2014). Hip Fracture: A Trigger for Palliative Care in Vulnerable Older Adults. JAMA Intern. Med..

[7] Balasegaram S., Majeed A., Fitz-Clarence H. (2001). Trends in Hospital Admissions for Fractures of the Hip and Femur in England, 1989–1990 to 1997–1998. J. Public Health Med..

[8] Boufous S., Finch C.F., Lord S.R. (2004). Incidence of Hip Fracture in New South Wales: Are Our Efforts Having an Effect?. Med. J. Aust..

[9] Cicvarić T., Bencević-Striehl H., Juretić I., Marinović M., Grzalja N., Ostrić M. (2010). Hip Fractures in Elderly—Ten Years Analysis. Coll. Antropol..

[10] Hagino H., Osaki M., Okuda R., Enokida S., Nagashima H. (2020). Recent Trends in the Incidence of Hip Fracture in Tottori Prefecture, Japan: Changes over 32 Years. Arch. Osteoporos..

[11] Langley J., Samaranayaka A., Davie G., Campbell A.J. (2011). Age, Cohort and Period Effects on Hip Fracture Incidence: Analysis and Predictions from New Zealand Data 1974–2007. Osteoporos. Int..

[12] Lehtonen E.J.I., Stibolt R.D., Smith W., Wills B., Pinto M.C., McGwin G., Shah A., Godoy-Santos A.L., Naranje S. (2018). Trends in Surgical Treatment of Femoral Neck Fractures in the Elderly. Einstein.

[13] Requena G., Abbing-Karahagopian V., Huerta C., De Bruin M.L., Alvarez Y., Miret M., Hesse U., Gardarsdottir H., Souverein P.C., Slattery J. (2014). Incidence Rates and Trends of Hip/Femur Fractures in Five European Countries: Comparison Using e-Healthcare Records Databases. Calcif. Tissue Int..

[14] Stephenson S., Langley J., Campbell J., Gillespie W. (2003). Upward Trends in the Incidence of Neck of Femur Fractures in the Elderly. N. Z. Med. J..

[15] Wilk R., Skrzypek M., Kowalska M., Kusz D., Koczy B., Zagórski P., Pluskiewicz W. (2018). The 13-Year Observation of Hip Fracture in Poland-Worrying Trend and Prognosis for the Future. Aging Clin. Exp. Res..

[16] Ju D.G., Rajaee S.S., Mirocha J., Lin C.A., Moon C.N. (2017). Nationwide Analysis of Femoral Neck Fractures in Elderly Patients: A Receding Tide. J. Bone Joint Surg. Am..

[17] Miller B.J., Lu X., Cram P. (2013). The Trends in Treatment of Femoral Neck Fractures in the Medicare Population from 1991 to 2008. J. Bone Joint Surg. Am..

[18] Gehlbach S.H., Avrunin J.S., Puleo E. (2007). Trends in Hospital Care for Hip Fractures. Osteoporos. Int..

[19] Leslie W.D., O’Donnell S., Jean S., Lagacé C., Walsh P., Bancej C., Morin S., Hanley D.A., Papaioannou A. (2009). Osteoporosis Surveillance Expert Working Group Trends in Hip Fracture Rates in Canada. JAMA.

[20] Wang Z., Bhattacharyya T. (2011). Trends in Incidence of Subtrochanteric Fragility Fractures and Bisphosphonate Use among the US Elderly, 1996–2007. J. Bone Miner. Res..

[21] MacKinlay K., Falls T., Lau E., Day J., Kurtz S., Ong K., Malkani A. (2014). Decreasing Incidence of Femoral Neck Fractures in the Medicare Population. Orthopedics.

[22] Brauer C.A., Coca-Perraillon M., Cutler D.M., Rosen A.B. (2009). Incidence and Mortality of Hip Fractures in the United States. JAMA.

[23] Araujo T.P.F., Guimaraes T.M., Andrade-Silva F.B., Kojima K.E., Silva J.D.S. (2014). Influence of Time to Surgery on the Incidence of Complications in Femoral Neck Fracture Treated with Cannulated Screws. Injury.

[24] Major L.J., North J.B. (2016). Predictors of Mortality in Patients with Femoral Neck Fracture. J. Orthop. Surg..

[25] Mabry S.E., Cichos K.H., McMurtrie J.T., Pearson J.M., McGwin G., Ghanem E.S. (2019). Does Surgeon Fellowship Training Influence Outcomes in Hemiarthroplasty for Femoral Neck Fracture?. J. Arthroplast..

[26] von Elm E., Altman D.G., Egger M., Pocock S.J., Gøtzsche P.C., Vandenbroucke J.P. (2007). STROBE Initiative The Strengthening the Reporting of Observational Studies in Epidemiology (STROBE) Statement: Guidelines for Reporting Observational Studies. Ann. Intern. Med..

[27] Popolazione Residente al 1° Gennaio. http://dati.istat.it/Index.aspx?QueryId=18460.

[28] Linee Guida per La Qualità Delle Statistiche Del Sistema Statistico Nazionale. https://www.istat.it/it/files/2018/08/Linee-Guida-2.5-agosto-2018.pdf.

[29] LeLaurin J.H., Shorr R.I. (2019). Preventing Falls in Hospitalized Patients: State of the Science. Clin. Geriatr. Med..

[30] Hempel S., Newberry S., Wang Z., Booth M., Shanman R., Johnsen B., Shier V., Saliba D., Spector W.D., Ganz D.A. (2013). Hospital Fall Prevention: A Systematic Review of Implementation, Components, Adherence, and Effectiveness. J. Am. Geriatr. Soc..

[31] Campani D., Caristia S., Amariglio A., Piscone S., Ferrara L.I., Barisone M., Bortoluzzi S., Faggiano F., Dal Molin A., IPEST Working Group (2020). Home and Environmental Hazards Modification for Fall Prevention among the Elderly. Public Health Nurs..

[32] Rossini M., Adami S., Bertoldo F., Diacinti D., Gatti D., Giannini S., Giusti A., Malavolta N., Minisola S., Osella G. (2016). Guidelines for the Diagnosis, Prevention and Management of Osteoporosis. Reumatismo.

[33] Burge R., Dawson-Hughes B., Solomon D.H., Wong J.B., King A., Tosteson A. (2007). Incidence and Economic Burden of Osteoporosis-Related Fractures in the United States, 2005–2025. J. Bone Miner. Res..

[34] Hagino H., Furukawa K., Fujiwara S., Okano T., Katagiri H., Yamamoto K., Teshima R. (2009). Recent Trends in the Incidence and Lifetime Risk of Hip Fracture in Tottori, Japan. Osteoporos. Int..

[35] Switzer J.A., O’Connor M. (2021). American Academy of Orthopaedic Surgeons Management of Hip Fractures in Older Adults Evidence-Based Clinical Practice Guideline. J. Am. Acad. Orthop. Surg..

[36] Cram P., Yan L., Bohm E., Kuzyk P., Lix L.M., Morin S.N., Majumdar S.R., Leslie W.D. (2017). Trends in Operative and Nonoperative Hip Fracture Management 1990-2014: A Longitudinal Analysis of Manitoba Administrative Data. J. Am. Geriatr. Soc..

[37] Stronach B.M., Bergin P.F., Perez J.L., Watson S., Jones L.C., McGwin G., Ponce B.A. (2020). The Rising Use of Total Hip Arthroplasty for Femoral Neck Fractures in the United States. Hip Int..

[38] Hongisto M.T., Pihlajamäki H., Niemi S., Nuotio M., Kannus P., Mattila V.M. (2014). Surgical Procedures in Femoral Neck Fractures in Finland: A Nationwide Study between 1998 and 2011. Int. Orthop..

[39] Harris I.A., Cuthbert A., de Steiger R., Lewis P., Graves S.E. (2019). Practice Variation in Total Hip Arthroplasty versus Hemiarthroplasty for Treatment of Fractured Neck of Femur in Australia. Bone Joint J..

[40] Lee Y.-K., Ha Y.-C., Park C., Koo K.-H. (2013). Trends of Surgical Treatment in Femoral Neck Fracture: A Nationwide Study Based on Claim Registry. J. Arthroplast..

[41] Bishop J., Yang A., Githens M., Sox A.H.S. (2016). Evaluation of Contemporary Trends in Femoral Neck Fracture Management Reveals Discrepancies in Treatment. Geriatr. Orthop. Surg. Rehabil..

[42] Ekhtiari S., Gormley J., Axelrod D.E., Devji T., Bhandari M., Guyatt G.H. (2020). Total Hip Arthroplasty Versus Hemiarthroplasty for Displaced Femoral Neck Fracture: A Systematic Review and Meta-Analysis of Randomized Controlled Trials. J. Bone Joint Surg. Am..

[43] Roberts K.C., Brox W.T. (2015). AAOS Clinical Practice Guideline: Management of Hip Fractures in the Elderly. JAAOS J. Am. Acad. Orthop. Surg..

[44] Hochfelder J.P., Khatib O.N., Glait S.A., Slover J.D. (2014). Femoral Neck Fractures in New York State. Is the Rate of THA Increasing, and Do Race or Payer Influence Decision Making?. J. Orthop. Trauma.

[45] Cree M., Yang Q., Scharfenberger A., Johnson D., Carrière K.C. (2002). Variations in Treatment of Femoral Neck Fractures in Alberta. Can. J. Surg..

[46] OECD (2017). Average Length of Stay in Hospitals.

[47] Bottle A., Aylin P. (2006). Mortality Associated with Delay in Operation after Hip Fracture: Observational Study. BMJ.

[48] Bretherton C.P., Parker M.J. (2015). Early Surgery for Patients with a Fracture of the Hip Decreases 30-Day Mortality. Bone Jt. J..

[49] Gundel O., Thygesen L.C., Gögenur I., Ekeloef S. (2020). Postoperative Mortality after a Hip Fracture over a 15-Year Period in Denmark: A National Register Study. Acta Orthop..

[50] Wu T.Y., Hu H.Y., Lin S.Y., Chie W.C., Yang R.S., Liaw C.K. (2017). Trends in Hip Fracture Rates in Taiwan: A Nationwide Study from 1996 to 2010. Osteoporos. Int..

[51] Rajeev A., Ali M., Tuinebreijer W., Zourob E., Anto J. (2021). Preexisting Dementia Is Associated with Higher Mortality Rate in Patients with Femoral Neck Fracture. Aging Med..

[52] Du M.-J., Lin Y.-H., Chen W.-T., Zhao H. (2022). Advances in the Application of Ultrasound for Fracture Diagnosis and Treatment. Eur. Rev. Med. Pharmacol. Sci..

[53] Tornetta P., Kain M.S.H., Creevy W.R. (2007). Diagnosis of Femoral Neck Fractures in Patients with a Femoral Shaft Fracture. Improvement with a Standard Protocol. J. Bone Jt. Surg. Am..

[54] Saarenpää I., Partanen J., Jalovaara P. (2002). Basicervical Fracture—A Rare Type of Hip Fracture. Arch. Orthop. Trauma Surg..

