# Supplementary material for: Epidemiology and Management of Proximal Femoral Fractures in Italy between 2001 and 2016 in Older Adults: Analysis of the National Discharge Registry
Source: Int J Environ Res Public Health. 2022 Dec 17;19(24):16985. doi: 10.3390/ijerph192416985 (PMC9778915; doi:10.3390/ijerph192416985)
Supplement: Supplementary file 1 [file ijerph-19-16985-s001.zip › supplementary Table S1_new.pdf]

SUBJECTS AT RISK																		
Age category	2001	2002	2003	2004	2005	2006	2007	2008	2009	2010	2011	2012	2013	2014	2015	2016	Total per age category	age-specific weigth
65-69	3073473	3110160	3175010	3245826	3305695	3330888	3310485	3248401	3163189	3052238	3132885	3266751	3447791	3561371	3561371	3684918	52670452	0,271
70-74	2806332	2827563	2845138	2868206	2855635	2860272	2895708	2963744	3035304	3102183	3079669	3067340	3044129	2962274	2962274	2856226	47031997	0,242
75-79	2276531	2318298	2342508	2367105	2422471	2461918	2477023	2500298	2530250	2533595	2501983	2548841	2645596	2717980	2717980	2779553	40141930	0,207
80-84	1267135	1443173	1617294	1742300	1763978	1795994	1830380	1855159	1882772	1941292	1943792	1967415	2013904	2047054	2047054	2054494	29213190	0,151
85-89	830535	762848	685613	664383	738158	842446	957772	1071115	1147892	1171062	1173319	1204887	1235881	1264039	1264039	1305410	16319399	0,084
90-94	331701	362692	379956	399042	405204	390675	354223	318488	312948	362732	410027	466302	518906	555492	555492	561890	6685770	0,034
95-99	63292	69487	75195	83388	91040	99062	107807	114220	119141	122290	114118	101903	90851	91769	91769	108498	1543830	0,008
100+	5650	7108	7767	9091	10154	11497	12588	13733	14974	16145	15029	16390	17884	19095	19095	18765	214965	0,001
Population per year	10654649	10901329	11128481	11379341	11592335	11792752	11945986	12085158	12206470	12301537	12370822	12639829	13014942	13219074	13219074	13369754	193821533	
EVENTS																		
Age category	2001	2002	2003	2004	2005	2006	2007	2008	2009	2010	2011	2012	2013	2014	2015	2016	Total events per age category	
65-69	4726	4714	4698	4962	4987	5006	5001	5004	4845	4559	4206	4499	4591	4578	5058	5088	76522	
70-74	9305	9432	8924	9568	9495	9226	9004	9094	8985	8692	8570	8738	8483	8248	8125	7847	141736	
74-79	16421	16052	16006	16423	17131	17015	16958	17208	17063	16180	15391	15450	15132	15106	15440	15470	258446	
80-84	16709	19266	21615	23753	24953	25228	25477	25965	25516	25264	24310	24559	24163	24167	24405	23877	379227	
85-89	19871	18812	16724	15400	16538	17943	20957	23975	26514	26409	26182	26740	26491	26424	27152	27384	363516	
90-94	11643	12226	13054	12339	13358	12833	11904	11190	9814	10746	11587	13529	14724	16339	17027	16952	209265	
95-99	2703	2941	3179	2782	3144	3457	3606	3942	4067	4170	3995	3598	3287	2898	3366	3870	55005	
100+	270	320	339	234	289	329	353	361	377	453	457	491	530	537	575	510	6425	
Events per year	81648	83763	84539	85461	89895	91037	93260	96739	97181	96473	94698	97604	97401	98297	101148	100998	1490142	
Raw incidence (x 100,000 subjects)	766,3134	768,3742	759,6634	751,0189	775,4693	771,9742	780,6806	800,4777	796,1434	784,2353	765,4948	772,194	748,3783	743,5997	765,1671	755,4215	768,8216974	
AGE ADJUSTED EVENTS PER 100,000 PERSONS																		

Age category	2001	2002	2003	2004	2005	2006	2007	2008	2009	2010	2011	2012	2013	2014	2015	2016	Mean age-adjusted incidence per age category	
65-69	41,78586	41,18811	40,20989	41,54287	40,99601	40,84095	41,05162	41,8613	41,62303	40,58974	36,48294	37,42527	36,18523	34,93201	38,59461	37,52185	39,48064945	
70-74	80,45791	80,94367	76,11103	80,94727	80,6833	78,27039	75,45224	74,45702	71,83023	67,98979	67,52556	69,12601	67,62041	67,56392	66,55636	66,66571	73,12706581	
75-79	149,3903	143,4023	141,5135	143,6915	146,4604	143,1379	141,7884	142,5394	139,6652	132,2628	127,4027	125,54	118,4593	115,1064	117,6514	115,2687	133,3422536	
80-84	198,7488	201,2103	201,4388	205,4814	213,2095	211,7166	209,7896	210,9522	204,264	196,15	188,5003	188,1446	180,8377	177,9387	179,6911	175,1668	195,6578271	
85-89	201,4484	207,6343	205,3824	195,1661	188,6409	179,3309	184,2337	188,4624	194,4806	189,8778	187,8836	186,8604	180,4778	176,0112	180,8604	176,625	187,5519166	
90-94	121,0786	116,2775	118,5113	106,6623	113,7148	113,3084	115,9219	121,1955	108,1741	102,1905	97,4783	100,0801	97,87832	101,4605	105,7328	104,0684	107,9678799	
95-99	34,0169	33,71234	33,67433	26,57364	27,50729	27,79648	26,64258	27,48982	27,19007	27,1608	27,88432	28,12365	28,81824	25,15358	29,21565	28,41101	28,37919975	
100+	5,300063	4,993077	4,840744	2,854762	3,15665	3,173782	3,110167	2,915463	2,792346	3,111902	3,372499	3,322525	3,286828	3,119036	3,33975	3,014306	3,314905161	
Age-adjusted incidence per year (x 100,000 subjects)	832,2269	829,3616	821,6821	802,9198	814,3689	797,5753	797,9903	809,8731	790,0196	759,3332	736,5303	738,6225	713,5639	701,2853	721,6421	706,7418	768,8216974	

Weight: Total population per age category/ sum of (Total population per age category)

Raw incidence: population per year/events per year * 100,000

Age-adjusted incidence per year: column sum of ((number of events per year per age category/subjects at risk per year per age category)*age-specific weigth*100,000 subjects)
