# Supplementary material for: Epidemiology and Management of Proximal Femoral Fractures in Italy between 2001 and 2016 in Older Adults: Analysis of the National Discharge Registry
Source: Int J Environ Res Public Health. 2022 Dec 17;19(24):16985. doi: 10.3390/ijerph192416985 (PMC9778915; doi:10.3390/ijerph192416985)
Supplement: Supplementary file 1 [file ijerph-19-16985-s001.zip › Supplementary Table S2_new.pdf]

Supplementary Table S2. Type of fracture based on age and gender.

TOTAL													
	82001	82002	82003	82011	82012	82013	82020	82021	82022	82030	82031	82032	unspecified
65-69	13455 (17,6%)	10088 (13,2%)	6638 (8,7%)	111 (0,1%)	416 (0,5%)	228 (0,3%)	20943 (27,4%)	6959 (9,1%)	4327 (5,7%)	268 (0,4%)	206 (0,3%)	182 (0,2%)	12701 (16,6%)
70-74	23534 (16,6%)	17448 (12,3%)	11954 (8,4%)	153 (0,1%)	729 (0,5%)	366 (0,3%)	41944 (29,6%)	13929 (9,8%)	7789 (5,5%)	502 (0,4%)	387 (0,3%)	304 (0,2%)	22697 (16%)
74-79	39179 (15,2%)	29319 (11,3%)	21479 (8,3%)	256 (0,1%)	1245 (0,5%)	690 (0,3%)	83664 (32,4%)	27612 (10,7%)	13724 (5,3%)	932 (0,4%)	710 (0,3%)	509 (0,2%)	39127 (15,1%)
80-84	54486 (14,4%)	40836 (10,8%)	30065 (7,9%)	365 (0,1%)	1681 (0,4%)	984 (0,3%)	130596 (34,4%)	43935 (11,6%)	20093 (5,3%)	1318 (0,3%)	1208 (0,3%)	685 (0,2%)	52975 (14%)
85-89	48371 (13,3%)	37201 (10,2%)	28085 (7,7%)	341 (0,1%)	1588 (0,4%)	895 (0,2%)	131509 (36,2%)	45702 (12,6%)	18379 (5,1%)	1294 (0,4%)	1254 (0,3%)	626 (0,2%)	48271 (13,3%)
90-94	26100 (12,5%)	20422 (9,8%)	16721 (8%)	179 (0,1%)	868 (0,4%)	491 (0,2%)	77621 (37,1%)	27587 (13,2%)	10323 (4,9%)	685 (0,3%)	840 (0,4%)	393 (0,2%)	27035 (12,9%)
95-99	6634 (12,1%)	5337 (9,7%)	4399 (8%)	50 (0,1%)	218 (0,4%)	124 (0,2%)	20805 (37,8%)	7430 (13,5%)	2641 (4,8%)	181 (0,3%)	217 (0,4%)	102 (0,2%)	6867 (12,5%)
+99	749 (11,7%)	596 (9,3%)	524 (8,2%)	3 (0%)	26 (0,4%)	6 (0,1%)	2417 (37,6%)	961 (15%)	295 (4,6%)	19 (0,3%)	31 (0,5%)	12 (0,2%)	786 (12,2%)
MALES													
65-69	3341 (14,1%)	2923 (12,3%)	1991 (8,4%)	35 (0,1%)	107 (0,5%)	72 (0,3%)	7214 (30,5%)	2472 (10,4%)	1547 (6,5%)	103 (0,4%)	57 (0,2%)	75 (0,3%)	3753 (15,8%)
70-74	5705 (14,6%)	4654 (11,9%)	3357 (8,6%)	50 (0,1%)	191 (0,5%)	104 (0,3%)	12218 (31,3%)	4140 (10,6%)	2321 (5,9%)	152 (0,4%)	116 (0,3%)	94 (0,2%)	5942 (15,2%)
74-79	9329 (14,5%)	7438 (11,6%)	5484 (8,5%)	60 (0,1%)	307 (0,5%)	188 (0,3%)	20745 (32,3%)	6967 (10,9%)	3294 (5,1%)	253 (0,4%)	165 (0,3%)	137 (0,2%)	9792 (15,3%)
80-84	13056 (14,9%)	10021 (11,4%)	7287 (8,3%)	102 (0,1%)	417 (0,5%)	257 (0,3%)	28930 (33%)	9787 (11,2%)	4149 (4,7%)	320 (0,4%)	282 (0,3%)	125 (0,1%)	12943 (14,8%)

85-89	11877 (15%)	9176 (11,6%)	6467 (8,2%)	91 (0,1%)	365 (0,5%)	218 (0,3%)	26327 (33,2%)	9292 (11,7%)	3387 (4,3%)	312 (0,4%)	269 (0,3%)	99 (0,1%)	11439 (14,4%)
90-94	6257 (14,6%)	5001 (11,6%)	3642 (8,5%)	43 (0,1%)	198 (0,5%)	124 (0,3%)	14360 (33,4%)	5081 (11,8%)	1694 (3,9%)	143 (0,3%)	158 (0,4%)	76 (0,2%)	6174 (14,4%)
95-99	1512 (14,5%)	1214 (11,6%)	948 (9,1%)	11 (0,1%)	57 (0,5%)	17 (0,2%)	3442 (32,9%)	1271 (12,1%)	417 (4%)	34 (0,3%)	36 (0,3%)	12 (0,1%)	1492 (14,3%)
+99	175 (16,2%)	133 (12,3%)	87 (8,1%)	1 (0,1%)	8 (0,7%)	1 (0,1%)	352 (32,6%)	107 (9,9%)	42 (3,9%)	3 (0,3%)	5 (0,5%)	4 (0,4%)	161 (14,9%)
FEMALES													
65-69	10114 (19,1%)	7165 (13,6%)	4647 (8,8%)	76 (0,1%)	309 (0,6%)	156 (0,3%)	13729 (26%)	4487 (8,5%)	2780 (5,3%)	165 (0,3%)	149 (0,3%)	107 (0,2%)	8948 (16,9%)
70-74	17829 (17,4%)	12794 (12,5%)	8597 (8,4%)	103 (0,1%)	538 (0,5%)	262 (0,3%)	29726 (28,9%)	9789 (9,5%)	5468 (5,3%)	350 (0,3%)	271 (0,3%)	210 (0,2%)	16755 (16,3%)
74-79	29850 (15,4%)	21881 (11,3%)	15995 (8,2%)	196 (0,1%)	938 (0,5%)	502 (0,3%)	62919 (32,4%)	20645 (10,6%)	10430 (5,4%)	679 (0,3%)	545 (0,3%)	372 (0,2%)	29335 (15,1%)
80-84	41430 (14,2%)	30815 (10,6%)	22778 (7,8%)	263 (0,1%)	1264 (0,4%)	727 (0,2%)	101666 (34,9%)	34148 (11,7%)	15944 (5,5%)	998 (0,3%)	926 (0,3%)	560 (0,2%)	40032 (13,7%)
85-89	36494 (12,8%)	28025 (9,9%)	21618 (7,6%)	250 (0,1%)	1223 (0,4%)	677 (0,2%)	105182 (37%)	36410 (12,8%)	14992 (5,3%)	982 (0,3%)	985 (0,3%)	528 (0,2%)	36831 (13%)
90-94	19843 (11,9%)	15421 (9,3%)	13079 (7,9%)	136 (0,1%)	670 (0,4%)	367 (0,2%)	63261 (38%)	22506 (13,5%)	8629 (5,2%)	542 (0,3%)	682 (0,4%)	317 (0,2%)	20861 (12,5%)
95-99	5122 (11,5%)	4123 (9,3%)	3451 (7,7%)	39 (0,1%)	161 (0,4%)	107 (0,2%)	17363 (39%)	6159 (13,8%)	2224 (5%)	147 (0,3%)	181 (0,4%)	90 (0,2%)	5375 (12,1%)
+99	574 (10,7%)	463 (8,7%)	437 (8,2%)	2 (0%)	18 (0,3%)	5 (0,1%)	2065 (38,6%)	854 (16%)	253 (4,7%)	16 (0,3%)	26 (0,5%)	8 (0,1%)	625 (11,7%)

Data are reported as absolute frequency and row percentage for each age class; 82001, closed upper transcervical fracture; 82002, closed midcervical fracture; 82003, closed basicervical fracture; 82011, open upper transcervical fracture; 82012, open midcervical fracture; 82013, basicervical fracture; 82020, closed trochanteric fracture; 82021, closed intratrochanteric fracture; 82022, closed subtrochanteric fracture; 82030, open trochanteric fracture; 82031, opem intratrochanteric fracture; 82032, open subtrochanteric fracture.
