# Supplementary material for: Epidemiology and Management of Proximal Femoral Fractures in Italy between 2001 and 2016 in Older Adults: Analysis of the National Discharge Registry
Source: Int J Environ Res Public Health. 2022 Dec 17;19(24):16985. doi: 10.3390/ijerph192416985 (PMC9778915; doi:10.3390/ijerph192416985)
Supplement: Supplementary file 1 [file ijerph-19-16985-s001.zip › Supplementary Table S3.pdf]

Supplementary Table S3. Therapeutic management in different types of fracture and age categories.

	HA	THA	CRwIF	CRw/oIF	ORwIF	ORw/oIF	Other	Non-surgical
PERTROCHANTERIC								
65-69	399 (1,2%)	653 (2%)	4087 (12,4%)	161 (0,5%)	19908 (60,5%)	28 (0,1%)	992 (3%)	6657 (20,2%)
70-74	1102 (1,7%)	1029 (1,6%)	8231 (12,7%)	329 (0,5%)	39347 (60,7%)	74 (0,1%)	1716 (2,6%)	13027 (20,1%)
74-79	2459 (1,9%)	1593 (1,3%)	16578 (13%)	624 (0,5%)	77710 (61,1%)	118 (0,1%)	3101 (2,4%)	24969 (19,6%)
80-84	4184 (2,1%)	1620 (0,8%)	27148 (13,7%)	859 (0,4%)	122600 (62%)	166 (0,1%)	4405 (2,2%)	36854 (18,6%)
85-89	4164 (2,1%)	1172 (0,6%)	29237 (14,7%)	918 (0,5%)	123438 (62,1%)	163 (0,1%)	4014 (2%)	35660 (17,9%)
90-94	2519 (2,1%)	547 (0,5%)	18055 (15,4%)	617 (0,5%)	72670 (61,9%)	118 (0,1%)	2399 (2%)	20524 (17,5%)
95-99	680 (2,2%)	101 (0,3%)	4778 (15,2%)	177 (0,6%)	19163 (61,1%)	24 (0,1%)	596 (1,9%)	5857 (18,7%)
+99	73 (2%)	14 (0,4%)	611 (16,4%)	25 (0,7%)	2238 (59,9%)	6 (0,2%)	93 (2,5%)	675 (18,1%)
TRANSCERVICAL								
65-69	6191 (18,8%)	12910 (39,3%)	1404 (4,3%)	73 (0,2%)	5084 (15,5%)	7 (0%)	346 (1,1%)	4921 (15%)
70-74	16495 (25,4%)	19282 (29,7%)	1805 (2,8%)	141 (0,2%)	6978 (10,8%)	15 (0%)	547 (0,8%)	8921 (13,8%)
74-79	40790 (32,1%)	22074 (17,4%)	2442 (1,9%)	228 (0,2%)	10525 (8,3%)	15 (0%)	869 (0,7%)	15225 (12%)
80-84	70757 (35,8%)	17522 (8,9%)	3376 (1,7%)	334 (0,2%)	13770 (7%)	25 (0%)	1111 (0,6%)	21522 (10,9%)
85-89	68714 (34,6%)	10756 (5,4%)	3027 (1,5%)	372 (0,2%)	12699 (6,4%)	27 (0%)	1015 (0,5%)	19871 (10%)
90-94	37955 (32,3%)	4685 (4%)	1953 (1,7%)	283 (0,2%)	7380 (6,3%)	9 (0%)	615 (0,5%)	11901 (10,1%)
95-99	9331 (29,7%)	1102 (3,5%)	499 (1,6%)	84 (0,3%)	1993 (6,4%)	10 (0%)	156 (0,5%)	3587 (11,4%)
+99	994 (26,6%)	115 (3,1%)	70 (1,9%)	10 (0,3%)	261 (7%)	2 (0,1%)	14 (0,4%)	438 (11,7%)

Data are reported as absolute frequency and row percentage for each age category. HA, hemiarthroplasty; THA, total hip arthroplasty; CRwIF, closed reduction with internal fixation; CRw/oIF, closed reduction without internal fixation; ORwIF, open reduction with internal fixation; ORw/oIF, open reduction without internal fixation;
