# Supplementary material for: Epidemiology and Management of Proximal Femoral Fractures in Italy between 2001 and 2016 in Older Adults: Analysis of the National Discharge Registry
Source: Int J Environ Res Public Health. 2022 Dec 17;19(24):16985. doi: 10.3390/ijerph192416985 (PMC9778915; doi:10.3390/ijerph192416985)
Supplement: Supplementary file 1 [file ijerph-19-16985-s001.zip › Supplementary Table S4.pdf]

Supplementary Table S4. Incidence of different treatments during the study years in pertrochanteric and transcervical fractures.

PERTROCHANTERIC FRACTURES																
	2001	2002	2003	2004	2005	2006	2007	2008	2009	2010	2011	2012	2013	2014	2015	2016
HA	2,3	2,4	2,0	2,4	2,4	2,4	2,5	2,4	2,1	2,0	1,9	1,6	1,6	1,6	1,6	1,4
THA	0,8	0,9	0,9	1,0	1,0	1,1	1,2	1,0	0,9	0,8	0,8	0,8	0,8	0,7	0,7	0,7
CRwIF	16,6	13,8	13,2	12,7	12,4	12,2	11,2	11,7	11,9	13,0	13,8	14,9	15,6	16,2	17,1	17,3
ORwIF	54,3	57,5	59,6	61,1	60,4	61,0	59,7	58,8	59,6	63,6	64,2	64,1	65,0	64,7	64,5	64,6
Non Surgical	21,2	20,8	20,0	19,1	20,3	19,8	22,6	23,3	22,8	18,1	17,3	16,6	15,0	14,7	14,5	14,5
Other	4,7	4,5	4,4	3,6	3,4	3,4	2,8	2,8	2,7	2,5	2,0	2,1	2,0	2,1	1,7	1,5
TRANSCERVICAL FRACTURES																
HA	46,3	46,5	46,9	47,4	47,0	47,6	47,6	48,3	48,8	50,7	51,6	52,0	52,6	52,7	51,9	51,7
THA	13,2	15,0	16,6	17,2	17,3	18,4	18,6	17,6	18,0	18,3	17,7	17,9	17,4	17,8	18,9	19,1
CRwIF	4,3	3,6	3,1	2,8	2,7	2,6	2,2	2,3	2,3	2,5	2,5	2,8	3,0	3,2	3,3	3,7
ORwIF	13,6	13,6	13,0	12,9	11,9	12,1	11,4	11,7	11,0	11,2	10,8	11,0	10,8	10,8	11,1	10,5
Non Surgical	20,7	19,3	18,5	17,9	19,5	17,8	18,7	18,9	18,8	16,3	16,5	15,4	15,4	14,6	14,1	14,2
Other	2,0	1,9	1,9	1,8	1,6	1,6	1,5	1,2	1,1	1,0	0,9	0,8	0,8	0,9	0,8	0,8

Data are reported as incidence of treatment per 100 events. HA, hemiarthroplasty; THA, total hip arthroplasty; CRwIF, closed reduction with internal fixation; ORwIF, open reduction with internal fixation; “Other” includes open and closed reductions without internal fixation, other reductions and combinations of the other techniques.
